# Books and Authors

**Published:** 1908-04

**Authors:** 


### BOOKS AND AUTHORS.

---

**The Medical and Surgical Knowledge of William Shakespere.** With Explanatory Notes. By **John W. Wainwright,** M.D. Octavo, pp. 78. New York: Published by the author. 1907.

What a delightful literary work it must have been for this author to have prepared this work! The constant association with Shakespere's great creations for so long a time must of a truth be a charming occupation. In looking over the pages one cannot but be impressed with the great poet's familiarity with so much of medicine that betrays a knowledge of its actual science and art, much of which, indeed, is useful today. This is a volume that will afford agreeable entertainment for any and every physician of cultivated appetite for good literature. It will serve to beguile many a half hour,—those oases in the deserts of hard work, that occasionally will come to every doctor, no matter how busy. The author has interlarded the excerpts with explanatory notes, that make plainer obscure phrases; and this has been done in a spirit of modesty rather than off pedantry. Singularly enough, not only are the excerpts plentiful as applied to general medicine and surgery, but nearly all the specialties of the present day are represented. It would be an agreeable task to point these out in detail, but is not necessary. This book is valuable as an index to the text and will enable the interested searcher to find in the complete words of Shakespere more extended references which have a collateral bearing on the excerpts.

---

**Atlas and Textbook of Human Anatomy.** Volume III. By Professor **J. Sobotta,** of Wurzburg. Edited, with additions, by **J. Playfair McMurrich,** A.M., Ph.D., Professor of Anatomy at the University of Michigan, Ann Arbor. Quarto, 342 pages, containing 297 illustrations, mostly in colors. Philadelphia and London: W. B. Sanders Company. 1907. (Cloth, $6.00; half morocco, $7.00, net prices.)

This volume, the third, completes the series of an anatomical exposition, which must take rank amongst the better delineations of the subject. It is a surprisingly accurate treatise, and must of necessity become a favorite with teachers of human anatomy, the world over. The student, too, who would best inform himself and can afford the outlay, will surely obtain this admirable work. In this volume the vascular system, begun in the preceding one, is finished; the entire nervous system is depicted, and the organs of special sense are described. The text of Sobotta's work is interesting though scarcely an unnecessary or superfluous word is employed. In the older anatomical textbooks a dryness mostly prevailed; whereas the modern methods of teaching make this proverbial dry subject even fascinating. Now, about the illustrations—they fairly bristle with interest and gleam with color. One can almost see and feel the arteries pulsate, and see also the blood stream course through them. The nerves, too, seem to vibrate, their depiction is so realistic. The sense organs,—the eye and ear in particular,—are marvelously pictured in every detail and merit the careful study of the specialist. Finally, the price is so reasonable, so low in fact as to excite our special wonder.

---

**Diseases of Children for Nurses.** By **Robert S. McCombs,** M.D., 12 mo, pp. 431. Illustrated. Philadelphia and London: W. B. Saunders Co. (Cloth, $2.00.)

This author has taken up a new line of thought in addressing nurses,—namely, on the specialty of diseases of children. Some nurses seem particularly adapted to one line of nursing, others to another, and there would appear to be no good reason why those who prefer to nurse children should not have special instruction in that branch of nursing. But a nurse should first be taught the general subject, and afterward take up the particular work for which she may feel the better adapted. The text of this book is well prepared by a man of experience, the several groups are handled intelligently, and the special conditions are adequately outlined. Infant feeding, in particular, has received important attention and is handled with good judgment. The chapters are short, concise, and forceful, the diction being clear and the rhetoric pleasing without being ornate; in short, the aim of the author evidently has been to treat his subject scientifically without superfluous phraseology. The illustrations are such as help to explain the text, and not to make a mere picture book of the volume. We think nurses who devote their work to children will do well to obtain this book.

---

**The Sexual Instinct.** Its Use and Dangers as Affecting Heredity and Morals. By **James Foster Scott,** A.B., M.D., late Obstetrician to Columbia Hospital for Women, and Lying-in Asylum, Washington, D. C. Second edition. Small 8vo, p. 474. Illustrated. New York: E. B. Treat & Co. (Cloth, $2.00.)

It is about ten years since the first edition of this book appeared. Nothing new has been recorded in this edition. The most that can be said of it is that it is a "good book." It is an excellent homily for religious people to read, and is suited in particular for clergymen. They should familiarise themselves with it and then instruct their congregations in regard to its contents. It goes over ground that has been plowed and harrowed time and again, without yet yielding much of a crop. But we would not discourage a continuance of the effort on that account. It deals with a topic that is extremely difficult to handle, or to get results from no matter how capably the conditions may be delineated. We are not of those that believe much good can come from a semi- or quasi scientific discussion of the subject. It must be dealt with on the broad ground of medical science and the talk must be plain. Evils there are and they must be warned against in strong terms in order to be eradicated or even modified. These are our own views, and are given for whatever they may be worth. We think this book deserves a wide circulation which it does not seem to have attained heretofore, if two editions in ten years are reliable indications.

---

**A Textbook of Practical Gynecology.** By **D. Tod Gilliam**, M.D., Emeritus Professor of Gynecology in Starling-Ohio Medical College; Gynecologist to St. Anthony and St. Francis Hospitals. Second Edition. Illustrated with 350 engravings, a colored frontispiece, and 13 full-page half-tone plates. 642 royal octavo pages. F. A. Davis Company, Philadelphia. (Cloth, $4.50, net; half-morocco, $6.00, net.)

Four years have elapsed since the first edition of this work appeared. The changes made, as enumerated by the author, are chiefly relating to technics. We wrote approvingly of the first edition and in some detail. Now, we shall limit our observations to reaffirming our earlier opinion and a mention of the changes. Some of the text has been eliminated and new material introduced here and there to take its place. The author describes another operation for extensive cystocele, also one for prolapse of the uterus occurring after the menopause. A regional index of symptoms has been added with special reference to the needs of general practitioners and students; it is, however. so imperfect, so incomplete, that it does not add much value to the book. By and large, this is an excellent treatise, condensed, scientific, and practical, making it a safe guide on all the topics of which it treats.

---

**Surgical Applied Anatomy.** By **Sir Frederick Treves**, F.R.C.S., Sergeant-Surgeon to H.M. the King. (5th) edition. 12mo, 640 pages, 107 illustrations, of which 41 are in colors. Lea Brothers & Co., Philadelphia and New York. 1907. (Cloth, $2.25, net).

Sir Frederick Treves is too well-known as a surgeon, and his little book is too well known among surgeons to justify more than a passing notice in this place. The work, which is one of the series of manuals for students of medicine, has been revised with care throughout, in order to make it conform to the present date. In doing this the reviser has rewritten certain sections, and added considerable new material, thus making every effort to bring each chapter up to date. The illustrations, too, have received due attention, forty-three figures having been prepared. especially for this edition. The manual therefore becomes one of the most useful surgical guides for the student and junior practitioner.

---

**Modern Medicine.** Its Theory and Practice. In original contributions by American and Foreign authors. Edited by **William Osler**, M.D., Regius Professor of Medicine in Oxford University, Eng. Assisted by **Thomas McCrea**, M.D., Associate Professor of Medicine and Clinical Therapeutics in Johns Hopkins University Baltimore. In seven octavo volumes of about 900 pages each, illustrated. Volume III. Lea Brothers & Co., Philadelphia and New York, 1907. (Cloth, $6.00; leather, $7.00; half morocco, $7.50 net prices.)

The third volume of Osler's Modern Medicine follows closely along the lines laid down in the previous volumes as regards attention to detail and care in preparation. This section of the work concludes the infectious diseases and takes up diseases of the respiratory tract.

There is evidence a-plenty of careful attention to detail, and the arrangement of the subjects leaves nothing to be desired. Every infectious disease is most thoroughly considered and if, in some portions, there are lapses of judgment, it would be simple justice to ascribe it to acute Baltimoreitis and not carelessness.

For instance, in that section of the work devoted to the different phases of gonorrheal invasion, the treatment of conjunctivitis is suggested as consisting of "mild antiseptics," boracic acid and permanganate of potash, the latter 1-5000 in strength. Possibly in a less rarified atmosphere a silver preparation, such as argyrol or collargolum, might be of advantage to the patient. Otherwise, the suggestions of this section are of value. Speaking of cystitis the somewhat startling announcement is made that Young, of Baltimore, has shown that gonococci alone may cause it; that gonococci may be present in the bladder without causing cystitis, and that in chronic cystitis the same investigator has shown that gonococci may be the only infecting agent present. These discoveries will probably be of interest to Fuller of New York, also Guiteras of the same village; likewise a couple of members of the Keyes family, and a few other scattered but serious minded workers in the realm of genitourinary surgery.

There is one section of this portion of the book which has really a pathetic interest. That is where the susceptibility of children in hospitals to gonorrheal infection is mentioned. It is stated that nearly all large hospitals everywhere have had epidemics and continue to have them in the babies' wards; figures are given in connection with a New York institution; yet it does not appear that Johns Hopkins has ever had anything of the kind. On the surface it seems really too bad that the Baltimore institution should have been overlooked; it is about the only thing that has escaped it up to date.

Except for the slight discrepancies noted in passing, the article dealing with the gonorrheal infections is generally good; it is excellently written and is beyond doubt the most complete exposition of gonorrhea, as it may be seen in general practice, which has yet been published; and the author is congratulated on the very thorough manner in which he has presented the subject.

The fourth volume, now in press, will cover diseases of the circulatory system and blood; the fifth will be devoted to disease of the alimentary tract; the sixth is to group diseases of the kidneys, those associated with internal secretions, those of still obscure causation; the diseases of the muscles and vasomotor and trophic disorders. The seventh and final volume of the work will cover nervous and mental diseases.

**Essentials of Modern Electrotherapeutics.** An Elementary Textbook on the Scientific Therapeutic Use of Electricity and Radiant Energy. By **Frederick Finch Strong,** M.D. Instructor in Electrotherapeutics at Tufts College Medical School, Boston. New York: Rebman Company. (Price, $1.00).

Doubting that electricity has accomplished as much in therapeutics as some have been led to believe, we yet recognise the propriety of teaching the topic with scientific adequacy, and of publishing books that deal with it fairly. It is certain that, preying upon the credulity of the unwary, charlatans have made use of electricity to treat imaginary illnesses and to extract large sums from the pockets of their clientele without giving value received. This little book contains about all that is known on the subject, and it states it in a direct and concise manner. It is especially designed for students and is, of course, elementary in character. It is well illustrated with necessary instruments and appliances, and will prove interesting and instructive for all pursuing the topic of which it treats.

---

**Practice of Medicine for Nurses.** By **George Howard Hoxie,** M.D., Professor of Internal Medicine in the University of Kansas, Kansas City, Mo. 12 mo. pp. 284. Illustrated. Philadelphia and London: W. B. Saunders Company. 1908. (Cloth, $1.50.)

Amongst all the literature prepared for nurses,—and certainly there is a wealth of it,—this book should take rank with the best. It tells a nurse just enough of medicine, not too much which sometimes has been the case. It would be possible within appropriate limits to deal with all the diseases of the body, but this author has shown discretion not only in his selections, but in handling each one with conciseness. He has brought out the salient features of each, however, and he has told the nurse how to recognise the danger-line in those diseases where this becomes of importance. The nurse need not become a diagnostician, but she must know enough of medicine to become an intelligent helper to the physician, and she must faithfully carry out his instructions. We believe she will find this book of aid to her in many ways, and she will become a better nurse if she reads and heeds the information and advice given in its pages.

---

**Cosmetic Surgery.** The correction of featural imperfections. By **Charles C. Miller,** M.D. 136 pages. 73 illustrations. Published by the Author, 70 State St., Chicago, Ill. (Cloth, $1.50.)

It is remarkable how even very unsightly deformities of nose and face can be corrected nowadays without scar under the name "cosmetic surgery." The author of this monograph has set forth in a simple, though effective fashion, the usual procedures in featural surgery. The illustrations are many but in the main of indifferent quality. The price, too, is high for so small a brochure. Nevertheless, there is much of value in its pages and it will appeal in many ways to all interested in this branch of surgery.

---

**A Reference Handbook of Obstetric Nursing.** By **W. Reynolds Wilson,** M.D., Visiting Physician to the Philadelphia Lying-in Charity. 12 mo, pp. 258. Illustrated. Philadelphia and London: W. B. Saunders Company. (Limp leather, $1.75.)

We regard this handbook as the best ever issued on the topic with which it deals. The special points which makes for quality in its favor are, first, conciseness, not a superfluous paragraph being used. Second, the subtopics are well chosen, scarcely one could be omitted with propriety. Third, size and make-up of the book, which makes it convenient for the handbag or even the pocket. Fourth, the illustrations are excellent, being wood cuts from black and white drawings. Finally, its flexible cover, in red leather, its red edges, and its rounded corners, together with its hard finished paper and plain-faced type, make a handsome book. Every nurse who serves obstetric patients will do well to obtain this book and be guided by it.

---

**Physicians' Manual of the Pharmacopeia and the National Formulary.** An Epitome by **C. S. N. Hallenberg,** Ph.G., M.D., Professor of Pharmacy in the School of Pharmacy, University of Illinois.

This manual includes the names and a brief description of all the articles appearing in the U. S. Pharmacopeia and the National Formulary. There is included a therapeutic index and a list of common names of articles which cannot fail to be of use to physicians. The lines separating formulary and pharmacopeial articles is rather arbitrary in most instances, hence a manual covering both series is of decided value. The manual is published by the American Medical Association and is worth many times its cost, fifty cents.

---

**Syphilis in the Army.** By **Major H. C. French,** Royal Army Medical Corps. Small 8 vo, pp. 134. London: John Bale, Sons & Danielsson. (Cloth, $1.50 net.)

That this is an unusual book may be inferred by its title, but it will be understood to be such after it has been read. Major

French holds the unique appointment of War Office Specialist in Venereal Diseases and Dermatology, has served in India and Egypt, where ample opportunities were afforded for the study of these diseases. He has kept carefully prepared records of his clinical observations and the tables published are of great value because they are strictly accurate. The table of two hundred cases of syphilis treated at Cairo should be studied carefully as they indicate success on other than established lines. More attention than ever before is now being paid to prophylaxis, as well as treatment of venereal diseases, and opinions not only differ, but are being recasted or are undergoing changes. For all these reasons, Major French should receive respectful consideration, as his views will help to reach more accurate, more scientific, and more fixed or definite conclusions.

---

**Principles and Practice of Modern Otology.** By **John F. Barnhill,** M.D., Professor of Otology, Laryngology and Rhinology in Indiana University School of Medicine, and Ernest deWolfe Wales, B.S., M.D., Associate Professor of Otology, Laryngology and Rhinology in Indiana Univerity Medical School. Octavo, pp. 575. Illustrated. W. B. Saunders Co. (Cloth, $5.50.)

One of the principles underlying the preparation of this book was to modernise the practice of otology, and so well have the authors succeeded in this commendable effort that their work may properly be considered one of the best and most complete yet issued. Not only that, but it is interesting from a literary viewpoint. They also have endeavored to correct what they term "traditional beliefs," such as the senseless suggestion that children may outgrow aural ailments, and that a discharging ear is little more than an annoyance. They emphasise the supreme importance of careful examination and correct diagnosis, and their work on prophylaxis and treatment is most excellent and thorough.

The illustrations are beyond criticism, especially those descriptive of anatomic features and the presentation of the various instruments. The working directions for different operations are most comprehensive. In a word, there is little left to be desired in the book and it will be of especial value to the work-a-day physician who first sees, as a rule, the aural cases which, apparently simple, later become most serious and often destructive of physical well-being if not of life.

---

**A Treatise on Diseases of the Skin.** By **Henry W. Stelwagon,** M.D., Ph.D., Professor of Dermatology, Jefferson Medical College, Philadelphia. Fifth Edition. Octavo of 1.150 pages, with 267 text-illustrations, and 34 full-page colored and half-tone plates. Philadelphia and London: W. B. Saunders Company, 1907. (Cloth, $6.00; half morocco, $7.50 net prices.)

In this edition of a most excellent work all the obsolete and unnecessary material has been eliminated which means, in brief, that the book has been given a literary polishing up and made thoroughly modern as to text and illustration. In a way the author has followed the flag, for he has rewritten the articles on frambesia and oriental sore; much new material has been added to the articles dealing with verruga peruana and tinea imbricata, while entirely new are sections devoted to dhobie itch and uncinariol dermatitis—all affections which belong in the domain of tropical medicine.

For the first time, too, the eruptions of leukemia receive serious attention.

The classically exact illustrations need but an added word of commendation; additions have been made which are of distinct benefit.

---

**A Textbook of the Practice of Medicine.** By **James M. Anders,** M.D., Ph.D., LL.D., Professor of the Theory and Practice of Medicine and of Clinical Medicine, Medico-Chirurgical College, Philadelphia. Eighth Edition. Octavo of 1317 pages, fully illustrated. Philadelphia and London: W. B. Saunders Company, 1907. (Cloth, $5.50; half morocco, $7.00 net prices.)

One needs only to compare the latest edition of this most excellent and serviceable work with former volumes to note the rapid strides made in internal medicine. The book has been carefully revised and only the most recent, positive knowledge of internal medicine is presented. Much stress is laid upon diagnosis, differential diagnosis and treatment, the differential points and the illustrations particularly being elaborated.

Much space is given to preventive medicine, dietetics and physiologic and medicinal therapeutics. One feature which cannot fail to attract attention is the improvement of the classification of diseases.

Among the articles which have been entirely rewritten are those on ankylostomiasis, dracontiasis, trypanosomiasis and beriberi. Among the new subjects dealt with are: Parasitic infusoria, febrile tropical splenomegaly, aplastic anemia, *x*-ray in leukemia; polycythemia and cyanosis with splenic tumor; Stokes-Adams's disease; Vincent's angina, abortive pneumonia, and chronic appendicitis without preceding acute attack.

In its present form the book is in truth, a work on modern medicine.

---

**Diseases of the Nose and Throat.** By **D. Braden Kyle,** M.D., Professor of Laryngology and Rhinology, Jefferson Medical College, Philadelphia. Fourth Edition. Octavo, 725 pages, with 215 illustrations, 28 in colors. Philadelphia and London: W. B. Saunders Company, 1907. (Cloth, $4.00; half morocco, $5.50 net prices.)

In this new edition of Kyle's work the same general plan of arrangement of subjects as presented in the earlier editions has been followed, although the book as a whole has been entirely revised and brought up to date. There are a number of new articles which have not heretofore been presented, and they add to the completeness of the work. They include: Taking cold; lithemic rhinitis; chemic ulcers; fibromyoma of the nasopharynx; telangiectoma; syphilis of the septum; empyema of the antrum; bone cysts of the accessory sinuses; gangrene of the tonsil; actinomycosis of the tonsil; Vincent's angina; angioneurotic edema; pharyngeal aneurism; cough; congenital stridor; bronchoscopy; functional aphonia, and surgery of the larynx.

A novel feature of the book, and one which might be followed by writers in other branches, is that wherein the writer in making use of literature dealing with operations uses the quoted author's own words in order that his exact meaning would be given. The illustrations have been improved by the addition of several new plates.

